# Electroactive Phenolic Contributors and Antioxidant Capacity of Flesh and Peel of 11 Apple Cultivars Measured by Cyclic Voltammetry and HPLC–DAD–MS/MS

**DOI:** 10.3390/antiox9111054

**Published:** 2020-10-28

**Authors:** Danuta Zielińska, Marcin Turemko

**Affiliations:** Department of Chemistry, University of Warmia and Mazury in Olsztyn, Plac Lodzki 4, 10-727 Olsztyn, Poland; marcin.turemko@uwm.edu.pl

**Keywords:** apples, phenolic compounds, antioxidant, reducing and chelating capacity, cyclic voltammetry, HPLC–DAD–MS/MS

## Abstract

In this study, 11 apple cultivars were characterized by their total phenolic content (TPC) and total flavonoid content (TFC) and antioxidant, reducing, and chelating capacity by 2,2-diphenyl-1-picrylhydrazyl (DPPH) test, cyclic voltammetry (CV), and ferric reducing antioxidant power (FRAP) assays; and ferrous ion chelating capacity. The phenolic compounds in flesh and peel were determined by liquid chromatography coupled to mass spectrometry and diode array detector (HPLC–DAD–MS/MS) and their electroactivity by CV. The results showed higher TPC, TFC, and antioxidant capacity by DPPH test in the peels of all apple cultivars as compared to the respective flesh. The peel extracts also showed two-fold higher FRAP values as compared to the flesh extracts. The reducing capacity of the peel and flesh determined by CV measurements confirmed the results achieved by spectrophotometric methods of evaluating antioxidant capacity. There was no significant difference in chelating capacity in the peel and flesh. The HPLC–DAD–MS/MS analysis showed the presence of 11 phenolic compounds in the peel and flesh which varied in antioxidant, reducing, and chelating activity. The order of the phenolic compound content in flesh and peel in Quinte cultivar, which showed the highest antioxidant capacity, was as follows: epicatechin > chlorogenic acid > quercetin 3-arabinoside > quercetin 3-glucoside > cyanidin 3-galactoside > quercetin 3-rhamnoside > catechin > phloridzin > rutin > phloretin = quercetin. CV results were highly correlated with those obtained by spectrophotometry and HPLC–DAD–MS/MS, providing evidence to support the use of cyclic voltammetry as a rapid method to determine the phenolic profile and reducing the power of apple flesh and peel. The association between antioxidant assays and phenolic compound content showed that the highest contribution to the antioxidant capacity of apple peel and flesh was provided by catechin, epicatechin, and cyadinin-3-galactoside, while phloretin, phloridzin, and chlorogenic acid were the main contributors to chelating activity. Results from this study clearly indicate that removing the peel from apples may induce a significant loss of antioxidants.

## 1. Introduction

Apple is a popularly consumed fruit, mostly because of the pleasant taste and the fact that it is cultivated worldwide. Apples are a significant part of the human diet and are ranked in the top five consumed fruits in the world [1]. The beneficial health effects of apples have been ascribed to the polyphenolic compounds, a group of secondary plant metabolites, of which several thousand structurally different compounds have been identified [2,3]. Phenolic compounds are generally recognized as the main determinants of the biological activities of apples, such as the prevention of cardiovascular diseases, asthma and other lung dysfunctions, diabetes, obesity, and cancer [4,5,6,7] as well as age-related neurodegeneration [8,9]. The potential health benefits of polyphenols have been reviewed by Scalbert et al. [10]. Moreover, the content and composition of polyphenols present in apples are important because of their contribution to the sensory quality of fresh fruit and processed apple products [11].

Apples contain a variety of phenolic compounds that can be classified into five major sub-classes, with procyanidins being the most abundant class (between 40% and 89%), followed by hydroxycinnamic acids, dihydrochalcones, flavonols, anthocyanins, and flavan-3-ols [12]. Anthocyanins that contribute to the red color of apple fruits are exclusively found in the peel and represent less than 8% of total phenolics [13,14,15].

The flavan-3-ols can be found in the form of monomers, oligomers, and polymers (procyanidins), and flavonols are often associated with sugar moieties. The main sugars involved in glycosylation are galactose, glucose, rhamnose, arabinose, and xylose, and rutinose, a disaccharide, has also been found in apple. Dihydrochalcones are mainly associated with glucose and xyloglucose [16]. Moreover, the distribution and profile of phenolic compounds vary considerably among different cultivars, genotypes, ripening stages, and environmental conditions, and also within different tissues [13,15,17,18,19,20,21].

The antioxidant activity of polyphenolics has been studied extensively [22,23,24]. These compounds usually have a high redox potential, which allows them to act as reducing agents, hydrogen donors, and singlet oxygen quenchers [22]. Therefore, several methods to measure the antioxidant activity of polyphenols have been proposed and were recently reviewed [23,24]. Among other methods, scavenging stable radicals such as 2,2-diphenyl-1-picrylhydrazyl (DPPH) and 2,2’-azinobis(3-ethylbenzothiazoline-6-sulfonic acid) (ABTS), oxygen radical absorbance capacity (ORAC), total radical trapping antioxidant parameter (TRAP), ferric reducing antioxidant power (FRAP), and cupric ion (Cu^2+^) reducing power (CUPRAC) were employed in foods [23]. The determination of antioxidant activity by electrochemical methods is of increasing interest. Electrochemical methods used to determine antioxidant activity are still being developed. Among electrochemical techniques, the most widely used for this purpose is cyclic voltammetry (CV). In contrast to the aforementioned methods, electrochemical assays are low-cost and usually do not require time-consuming sample preparation. CV is based on an analysis of the anodic current (AC) waveform, which is a function of the reactive potential of a given compound in the sample. The CV tracing indicates the ability of a compound to donate electrons at the potential of the anodic wave. Therefore, in the past couple of years, CV has proven to be highly practical and efficient in determining the phenolic composition and reducing the power of complex matrices, including fruit extracts, honey, wine, tea, coffee, and kiwifruit [25,26,27,28,29]. Methods involving cyclic voltammetry (CV) have also been suggested as an instrument in evaluating the reducing activity of a wide spectrum of bioactive compounds and food extracts [25,30,31].

In many works, the content and antioxidant properties of polyphenols present in all parts of the apple fruit (skin, pulp, and seeds) were determined for various cultivars [32,33,34,35]. However, these You jumped the numbers in between.studies mainly focused on the relationship between antioxidant activity and total phenolic content, while a limited amount of data were available on phenolic profiles and their contribution to the antioxidant activity of apple extracts. Additionally, the correlation between the different antioxidant activity evaluation assays, chelating activity, and contents of individual apple polyphenols has not yet been fully investigated. However, the feasibility of electrochemical methods in determining the antioxidant activity in the phenolic compounds of apple and extracts from the peel and flesh samples is yet to be studied.

In this study, the antioxidant properties and major phenolic contributors present in the flesh and peel extracts of 11 apple cultivars from Poland were addressed. We attempted a novel approach by investigating the feasibility of applying cyclic voltammetry (CV) to determine the reducing activity of major phenolic compounds and predicting the antioxidant capacity of apple extract from peel and flesh. The aims of this study were as follows: (1) to determine the antioxidant capacity of apple flesh and peel by peel by cyclic voltammetry and spectrophotometric assays, (2) to determine the profiles of phenolic compounds in the flesh and peel of popular apple cultivars by sensitive liquid chromatography (HPLC) coupled to mass spectrometry (MS) using the electrospray ionization (ESI) and diode array detector (DAD) methodology, (3) to determine the antioxidant activity of the identified phenolic compounds by cyclic voltammetry (CV) and spectrophotometric assays, and (4) to show the relationship between the content of individual phenolic compounds and the antioxidant capacity of apple flesh and peel.

## 2. Materials and Methods

### 2.1. Chemicals and Reagents

Chlorogenic acid, gallic acid, rutin, quercetin, quercetin-3-*O*-glucoside, quercetin-3-*O*-rhamnoside, (-)-epicatechin, and cyanidin-3-*O*-galactoside were supplied by Extrasynthese (Genay, France). Quercetin-3-*O*-arabinoside, (+)-catechin, phloretin, phloridzin, and other compounds were obtained from Sigma-Aldrich (Munich, Germany); 2,2-diphenyl-1-picrylhydrazyl (DPPH), 2,4,6-tri(2-pyridyl)-s-triazine (TPTZ) and 6-hydroxy-2,5,7,8-tetramethylchroman-2-carboxylic acid (Trolox) were purchased from Sigma (Sigma Chemical Co., St. Louis, MO, USA). Folin–Ciocalteu’s reagent and others of reagent-grade quality were from POCh (Gliwice, Poland). Ultrapure water was purified with a Millipore Direct-Q UV 3 System (Bedford, MA, USA). Flavonoids and solvents were HPLC-grade quality, and other reagents were at least reagent-grade quality.

### 2.2. Sample and Standard Preparations

The studied material consisted of 11 first-quality grade apple cultivars at their ripe period of growth. Early varieties of apples such as Antonówka, Delikates, Early Geneva, Papierówka, Paulared, Sunrise, and Quinte were harvested in August, while Gloster, Jonagored, Ligol, and Rubinola cultivars, which are late varieties, were harvested in September, both during the 2019 season. All fruits were purchased from the Experimental and Production Institute “Pozorty” Sp. z o.o. in Olsztyn (Poland). Fruit samples (10 apple fruits randomly selected) were washed with distilled water to remove foreign substances and manually peeled using a hand peeler (1–2 mm thickness), cored, and cut into small pieces. The weighed apple flesh and peels were pooled separately. Before freeze drying (FD), the apple flesh and peels were frozen overnight at −25 °C and dried in the FreeZone 2.5 freeze dryer (Labconco, CA, USA). During FD, pressure was reduced to 16 Pa. The temperature in the drying chamber was −55 °C, and the shelf temperature was 26 °C. Apple flesh and peels were kept in the drying chamber for 24 h. The lyophilized samples were ground in a laboratory mill and stored at −20 °C up to further analysis. The moisture content of the peel from all apple cultivars ranged from 93.44 to 94.83%, and that of flesh was in the range from 91.63 to 93.01%. About 100 mg (for spectrophotometric methods) and 250 mg (for the electrochemical method) of lyophilized flesh and peel were extracted with 1 mL of 80% methanol by 30 s sonication. Then, the mixture was vortexed for 30 s, again sonicated and vortexed, and centrifuged for 5 min (13,200 rpm). This step was repeated five times and supernatant was collected in a 5 mL flask. Finally, all extracts were kept in dark-glass vials at −20 °C for further analysis.

### 2.3. Spectrophotometric Determination of Total Phenolic and Flavonoid Content

Total phenolic content (TPC) was determined according to the Folin–Ciocalteu (FC) assay as described by Shahidi and Naczk [36] with a slight modification. A volume of 90 µL of sample extract (10 mg/ml), 90 µL of FC reagent (diluted 1:10, *v*/*v*), 180 μL of saturated solution of Na_2_CO_3_, and 1440 μL of H_2_O were mixed and allowed to react for 25 min in a thermomixer (Comfort, Eppendorf) at room temperature. The absorbance was measured at 725 nm in a UV-1800 spectrophotometer (Shimadzu, Japan) with a temperature controller (TCC-Controller, Shimadzu, CA, USA). Catechin was used as a standard and the results were based on the standard curve equation of catechin (0.0625–1.0 mM) and expressed as a catechin equivalent (CAE) in μg/g of fresh weight. The measurements were made in triplicate.

Total flavonoid content (TFC) was determined based on the method by Jia et al. [37] with some modifications. A volume of 1230 μL of extract (10 mg/mL) was mixed with 62 μL of 5% NaNO_2_ solution (m/v). After incubation in the thermomixer (Comfort, Eppendorf) at room temperature for 6 min, 123 μL of 10% AlCl_3_·6H_2_O was added. Again, the mixture was incubated under the same conditions for 6 min, then 410 μL of 1M NaOH was added and the mixture was centrifuged for 5 min at 2000 rpm (Centrifuge 5424, Eppendorf). The absorbance of the reaction mixture was measured against the reagent blank at 510 nm with the UV-1800 spectrophotometer with a temperature controller (TCC-Controller, Shimadzu, city, State, country). Catechin was used as a standard and the results were based on standard curve equation of catechin (0.0625–0.5 mM) and expressed as catechin equivalent (CAE) in μg/g of fresh weight. The measurements were done in triplicate.

### 2.4. Analysis of Phenolic Compounds by HPLC–DAD–MS/MS

The identification of phenolic compounds was done by means of liquid chromatography (HPLC) coupled to mass spectrometry (MS) using the electrospray ionization interface (ESI). Quantification of phenolic compounds was carried out by using HPLC with diode array detector (DAD). The analysis of phenolic compounds was performed on a micro-HPLC system (LC200, Eksigent, Dublin, CA, USA) with pump, autosampler, column oven, and system controller. The micro-HPLC was connected in series to a QTRAP 5500 mass spectrometer (AB Sciex, Canada) equipped with a triple quadrupole, ion trap, and an ion source of electrospray ionization (ESI). The analytical column was a Halo C18 column (50 mm × 0.5 mm, 2.7 µm i.d.; Eksigent, Dublin, CA, USA). Eluent A was water/formic acid, 99.05/0.95 (*v*/*v*); eluent B was acetonitrile/formic acid, 99.05/0.95 (*v*/*v*). A gradient elution program was used: 5–5–90–90–5–5% B in 0–0.1–2–2.5–2.7–3 min. Before chromatographic analysis, apple extract was centrifuged (20 min, 13,000 *g*). Aliquot (2 µL) of sample solution was injected, with flow rate of 15 µL/min, at a column temperature of 45 °C. Phenolic compounds detected in the apple extracts were identified according to their MS/MS fragmentation spectrum (*m*/*z* values). Mass spectrometry data were obtained in positive- and negative-ion mode. The optimal identification of phenolic compounds was achieved under the following conditions: curtain gas: 25 L/min, collision gas: 9 L/min, ions pray voltage: 5400 V (for positive-ion mode) and −4500 V (for negative-ion mode), temperature: 350 °C, 1 ion source gas: 35 L/min and 2 ion source gas: 30 L/min; and entrance potential: 10 V (for positive-ion mode) and −10 V (for negative-ion mode). Phenolic compounds were quantified from the determined multiple reaction monitoring pairs (MRM) as shown in Table 1 and the calibration curves of external standards (range of 10–1000 nM).

### 2.5. Ferric Reducing Antioxidant Power (FRAP) Assay

The FRAP assay was carried out with some modifications according to the method of Benzie and Strain [38]. Briefly, the FRAP-2,4,6-tri(2-pyridyl)-s-triazine (TPTZ) reagent was prepared from the sodium acetate buffer (300 mM, pH 3.6), 10 mM 2,4,6-tri(2-pyridyl)-s-triazine (TPTZ) solution (40 mM HCl as solvent), and 20 mM FeCl_3_·6H_2_O in a volume ratio of 10:1:1. The FRAP reagent was freshly prepared on the day of the measurements. An aliquot of 75 μL of the extract was mixed with 225 μL of ultrapure water and 2250 μL of FRAP reagent (pre-incubated for 5 min at 25 °C). The absorbance of the reagent mixture was measured at 593 nm after 30 min incubation at 25 °C. Samples were measured in 3 replicates. The standard curve was prepared using Trolox solution (0.034–0.612 mM), and the results were expressed as μmol of Trolox equivalent per gram of apple fresh weight (μmol TE/g FW).

### 2.6. DPPH Assay

DPPH assay was based on the method of Brand-Williams et al. [39]. The samples were diluted to a proper concentration to make sure that the test results were readable between the absorbance values of 0.2–0.8. A volume of 100 µL of sample was mixed with 2 mL of methanol and then reacted with 250 µL of DPPH solution (10 mg DPPH in 25 mL of methanol) The reaction mixture was incubated in the dark at room temperature for 20 min, after which the absorbance at 517 nm was recorded. The test was performed in triplicate. The Trolox calibration curve (0.1–1.0 mM) was plotted as a function of the decrease in absorbance. The percentage of inhibition of DPPH radical by tested samples was calculated using the following equation, expressed as μmol TE/g FW:

Scavenging activity % = 100 − [(Abs. of sample − Abs. of blank) × 100/Abs. of control]

### 2.7. Cyclic Voltammetry (CV) Assay

Cyclic voltammograms were recorded using a Gamry G 750 potentiostat (Warminster, PA, USA). The working electrode was a 3 mm diameter glassy carbon disk electrode (BAS MF-2012). An Ag/AgCl reference electrode was used in conjunction with a platinum wire as a counter electrode. Given the effect of polyphenol adsorption on the voltammetric response, which was observed in our previous work [40], it was considered important to run the voltammograms in as consistent a manner as possible. The following electrode cleaning procedure was undertaken between each run. The electrode was thoroughly hand-polished with 0.05 μm alumina powder (BAS CF-1050) on the polishing cloth (BAS MF-1040) and rinsed thoroughly with Milli-Q grade water. Before taking the cyclic voltammogram of the test solution, a background cyclic voltammogram was run in the buffer solution in the potential range from −0.1 to 1.3 V at a scan rate of 0.1 V s^−1^, and the electrode was rinsed with Milli-Q grade water and methanol and dried. Apple extracts were diluted with Britton-Robinson (B-R) buffer (0.1 M, pH 7.4) at a 1:1 ratio, and the final extract concentration was 25 mg/mL. Cyclic voltammograms were taken in the potential range from −0.1 to 1.3 V at scan rate of 0.1 V s^−1^, with only the first scan being recorded. Background cyclic voltammograms were subtracted from those obtained for apple extracts to allow the oxidation and reduction processes to be more clearly revealed. For the purpose of testing, the total anodic peak wave area of the voltammograms was calculated within the range of 0 to 1.1 V. This method was based on the correlations between the total anodic peak wave area of a cyclic voltammogram and the antioxidant capacity of the sample and reference substance. For the reference, a solution of Trolox within the concentration range of 0.15–1.00 mM was used, and the results were expressed as μmol TE/g FW.

### 2.8. Chelating Activity on Ferrous Ions

The chelating activity of ferrous irons was measured by the inhibition of the formation of an Fe^2+^-ferrozine complex after treating the apple extracts with Fe^2+^ according to Mladénka et al. [41]. Briefly, 0.4 mL of apple extract (0.5 mg/mL) and 0.2 mL of HEPES (pH 7.5, 0.12 mM) were added to a solution of 0.4 mM FeSO_4_·7H_2_O (0.2 mL) and mixed for 2 min. Then, a volume of 800 µL of ferrozine solution (0.5 mM) was added and the absorbance of the ferrous ion–ferrozine complex was immediately measured at 562 nm. Ferrous ion solution was prepared daily and purged by argon 5.5 grade quality (Linde, Germany). For the comparison of ferrous chelating activity, deferoxamine (DEF) was used as a standard iron chelator. The amount of remaining ferrous ion was calculated from the difference of absorbance between the apple extract sample (with ferrozine) and its control blank (without ferrozine) was divided by the difference of the control sample (known amount of ferrous ion without apple extract) and its corresponding blank. A standard curve of Fe^2+^ ions was prepared within the range of concentration of 5–60 μM. The ferrous chelation efficiency of tested apple extract was expressed in %. Measurements were done at least in triplicate.

### 2.9. Analysis of Antioxidant Activity of Phenolic Compounds Identified by HPLC–DAD–MS/MS

The antioxidant activity of the phenolic compounds identified by HPLC–DAD–MS/MS in apple peel and flesh was provided by cyclic voltammetry and by spectrophotometric assays (DPPH, FRAP, and chelating activity). Stock solutions of each standard compound were also dissolved in 80% methanol (*v*/*v*, pH 6.0) and stored at −80 °C. Results were expressed as mM of Trolox of 9 independent experiments (*n* = 9). The ferrous chelation efficiency of apple phenolics was expressed in % of 9 replicates (*n* = 9).

### 2.10. Statistical Analyses

The analyses were performed in triplicate, and the results were displayed as the mean ± standard deviation (SD). The differences in identified phenolic contents in the peel and flesh of 11 apple cultivars were determined by one-way analysis of variance (ANOVA) with Fisher’s least significant difference test (*p* < 0.05). Correlations between the antioxidant capacity assays and polyphenol compounds were analyzed using the Pearson correlation coefficient test. All analyses were performed using Statistica software (v. 12; StatSoft, Tulsa, OK, USA). Statistical significance thresholds for correlations were set at *p*-value <  0.05 (*), *p*  <  0.01 (**) and *p*  <  0.001 (***).

## 3. Results and Discussion

### 3.1. Total Phenolic Content (TPC) and Total Flavonoid Content (TFC)

According to several authors, the phenolic and flavonoid contents vary among different cultivars of fruits and vegetables, and within different tissues [42,43]. With respect to this condition, for this study, the cultivars were selected in order to eliminate the impact of soil, fertilizing method, and climatic conditions on apples, because all fruits were grown exclusively in one orchard (Pozorty, Olsztyn). It can be supposed that the antioxidant activity of apples depends, to a large extent, on the cultivar. The 11 apple cultivars selected for this study were characterized by an over-color of the peel that ranged from green-yellow (Papierówka and Antonówka) to red (Paulared, Quinte, Gloster, and Rubinola). The distribution of polyphenol compounds between the peel and flesh of analyzed cultivars for total phenolic content (TPC) for total flavonoid content (TFC) is shown in Table 2.

For all the studied cultivars, both the TPC and TFC were higher in the apple peel extract than in the flesh extract. Furthermore, significant differences were found between the cultivars (*p* < 0.05) in TPC and TFC. TPC ranged between 1821.3 and 3278.6 µg CAE/g fresh peel (Table 2). Quinte had the highest TPC, followed closely by Early Geneva and Jonagored (3278.6, 3147, and 3123.1 µg CAE/g fresh peel, respectively), whereas Ligol and Antonówka had the lowest TPC (1821.3 and 2051.6 µg CAE/g fresh peel, respectively). The remaining cultivars were intermediate, with a TPC varying between 2194.7 and 2916.1 µg CAE/g fresh peel. However, the total phenolics were lower in flesh than in peel, ranging from 535.5 to 1740.3 µg CAE/g fresh flesh (Table 2). Ligol and Gloster presented low contents with less than 600 µg CAE/g fresh flesh, whereas Quinte showed a concentration level of 1740.3 µg CAE/g fresh flesh. TPC values for the flesh and peel extracts of the studied apple cultivars were comparable with those previously reported by Tsao et al. [13] and Carbone et al. [18]. Tsao et al. [13] reported, for the eight most popular apple cultivars grown in Ontario, that the TPC ranged from 1016.5 to 2350.4 µg GAE/g of FW in the peel and 177.4 to 933.6 µg GAE/g of FW in the flesh. These values were found to be in agreement with our TPC results obtained for the 11 tested apple cultivars (1821.3 to 3278.6 µg CAE/g FW and 535.5 to 1740.3 µg CAE/g FW for peel and flesh, respectively). In the present study, the flavonoid content (TFC) ranged from 25% to 44.7% of the TPC in the peel and from 28.9% to 61.0% in the flesh, and these results are in agreement with those reported by Carbone et al. [18].

Recent studies also have demonstrated the influence of the apple cultivar on the fruit’s phytochemical content [13,18] as well as a possible relationship between the color of different cultivars and their nutritional values [44]. In West Himalayan apple varieties, it was confirmed that there is a significant difference in phenolic content among cultivars and locations [45]. It was also confirmed that the variety and maturity of apples have a significant impact on chemical composition, the concentration of polyphenols, and level of antioxidant activity [35]. In a study of 120 apple varieties, a large diversity in polyphenolic compound content was observed depending on the variety [35]. According to this research, the highest phenolic content was found in Ozark Gold (~2116.03 mg/kg), and the lowest concentration was for Ligol (~814.17 mg/kg). The quality and quantity of polyphenols in apples directly influences their antioxidant activity [46].

While a TPC assay can adequately differentiate between apple cultivars that are high and low in polyphenols, it was less useful as a forecaster of potential health benefits. This is because the TPC measurements include nonabsorbable polymeric polyphenols as well as smaller, potentially absorbable polyphenolic compounds, which are thought to be mainly responsible for the observed physiological effects. Although some degradation products of polymeric polyphenols are absorbed in the colon, it is still not fully explained whether they have beneficial physiological effects. Whether the magnitude of polyphenol content has any relevance to the health properties of apples must then be tested by measuring the individual small molecular weight polyphenols.

### 3.2. Antioxidant and Chelating Capacity of Apple Flesh and Peel Determined by Spectrophotometric Assays

The antioxidant capacity of food should be evaluated with a variety of methods that can address the different mechanisms [47,48]. In the present study, spectrophotometric methods such as DPPH radical-scavenging activity and ferric reducing antioxidant power (FRAP) were used to determine the antioxidant capacity of apple extracts. The DPPH assay is based on a mixed mechanism of free radical DPPH^•^ stabilization: hydrogen atom transfer and electron transfer. This assay presents some critical analytical points [49], but it has the great advantage of being easy to use. The FRAP assay is based on the ability of antioxidants to reduce ferric(III) ions to ferrous(II) ions by the electron transfer mechanism. The antioxidant capacity of apple peel and flesh extracts, evaluated by DPPH and FRAP assays and expressed as Trolox equivalent (µmol TE/g FW), is shown in Table 2.

The results obtained in the DPPH assay showed that, in general, the flesh and peel have intermediate radical-scavenging activity, with peels being better scavengers than flesh. Apple peel extracts from Jonagored (8.65 µmol TE/g FW), Gloster (8.19 µmol TE/g FW), and Rubinola (7.91 µmol TE/g FW) had the highest and extracts from Papierówka (5.62 µmol TE/g FW) and Antonówka (5.27 µmol TE/g FW) showed the lowest free radical scavenging capacity among the tested apple cultivars. In the case of apple flesh extracts, the DPPH values ranged between 2.23 and 4.65 µmol TE/g FW for Gloster and Quinte cultivars, respectively. These results were consistent with those reported for different apple varieties by Carbone et al. [18]. A highly significant relationship was found between the DPPH antiradical activity of apple flesh extracts and the concentration of TPC (r = 0.96; *p* < 0.001) and TFC (r = 0.82; *p* < 0.01). Panzela et al. [50] also found a good correlation between the percentage of reduced DPPH and the concentration of total polyphenols (r = 0.79) and total flavan-3-ols (r = 0.77).

For all the tested apple cultivars, peel extracts had a much greater ferric-reducing antioxidant power than flesh extracts, with a two-fold difference (Table 2). Quinte and Jonagored peel extracts had the highest FRAP values (21.31 µmol TE/g FW and 20.89 µmol TE/g FW, respectively), and Antonówka and Ligol peel extracts had the lowest (12.73 µmol TE/g FW and 12.40 µmol TE/g FW, respectively). These results were consistent with the total polyphenol and total flavonoid concentration in Early Geneva, Quinte, and Jonagored and in Ligol. The FRAP activity of the extracts from different apple cultivars was positively correlated with both total phenolic and total flavonoid content (r = 0.9972 and 0.8229, respectively). These results were in agreement with the findings of Tsao et al. [13], who studied the total phenolic compounds of eight apple cultivars and also obtained a good correlation between the TPC and FRAP activity (r = 0.95).

The phenolic compounds of apple may act as reducing agents, hydrogen donors, free radical scavengers, and singlet oxygen quenchers, and may exhibit antioxidant activity via the chelation of metal ions [23]. In this study, ferrous ion chelating activity was measured by the inhibition of the formation of a Fe(II)–ferrozine complex after the treatment of peel and flesh extracts with Fe(II). The chelating capacity of apple peel ranged from 51.80 (Quinte cultivar) to 19.30% (Ligol cultivar), while in flesh it was within the range of 49.46 (Papierówka cultivar) to 18.31% (Ligol cultivar). The chelating activities of peels of individual apple cultivars were significantly different and the same observation was noted for apple flesh (*p* < 0.05).

### 3.3. Reducing Capacity of Apple Flesh and Peel Determined by Cyclic Voltammetry

**Figure 1:**
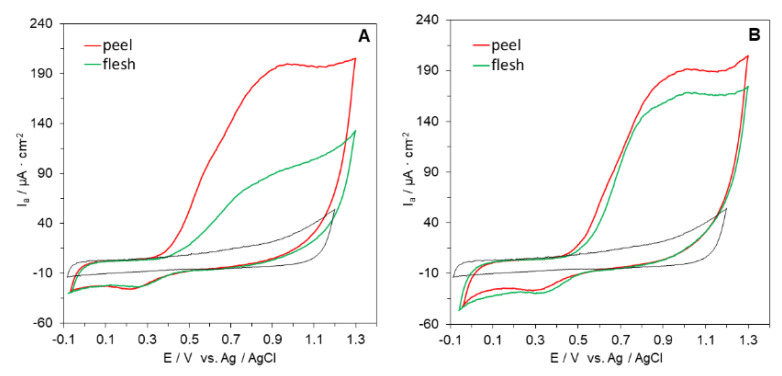
Cyclic voltammograms of peel and flesh extracts of selected apple cultivars: (**A**) Jonagored, (**B**) Antonówka. CV of electrolyte solution shown as dotted gray line. Operative conditions: extract concentration 25 mg/mL; pH 6.0; scan rate 0.1 V/s.

**Figure 2:**
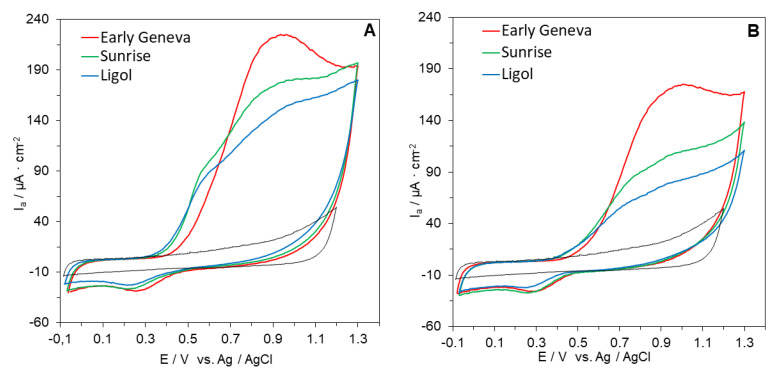
Cyclic voltammograms of selected apple cultivars: (**A**) extracts from peel; (**B**) extracts from flesh. CV of electrolyte solution shown as dotted gray line. Operative conditions: extract concentration 25 mg/mL; pH 6.0; scan rate 0.1 V/s.

In this study, we conducted a critical evaluation of the cyclic voltammetry method for the determination and rapid screening of the reducing capacity of peel and flesh of 11 apple cultivars compared with DPPH and FRAP assays. The representative cyclic voltammograms of peel and flesh extracts (25 mg/mL) were recorded from −0.1 to 1.3 mV at a scan rate of 100 mV/s (Figure 1 and Figure 2).

The obtained voltammograms show that the apple extracts exhibited well defined oxidation and reduction voltammetric peaks. The area of each voltammetric peak was related to the concentration of antioxidants. A broad anodic peak between 0.4 and 1.0 V was observed. This peak was due to the response of several antioxidants with different oxidation potentials, mainly flavonoids and phenolic acids. The results show that the samples contained multiple reducing agents in the respective extracts. Therefore, the area under the anodic current waveform (area under the curve, AUC) was taken to reflect the reducing capacity of the samples compared to a set of Trolox solutions, as suggested by Chevion et al. [51], Martinez et al. [52], and Zielińska and Zieliński [40]. This provides a marked advantage in some cases, particularly when the AUC wave represents more than a single component. Higher AUC indicates a higher reducing capacity of the investigated extract.

The reducing capacity of peels as shown by CV ranged from 6.80 to 4.35 μmol TE/g FW (Table 2). The highest reducing capacity was noted for Paulared, Jonagored, and Quinte cultivars and the lowest for Rubinola, Antonówka, and Ligol. In contrast, the reducing capacity of apple flesh was noted to be at least twice as low (Table 2), ranging from 3.95 (Quinte) to 1.44 (Ligol) μmol TE/g FW. The reducing capacity of peels as shown by CV was positively correlated with both the total phenolic content (r = 0.867; *p* < 0.01) and total flavonoid content (r = 0.752, *p* < 0.01). The same trend in correlation efficient values was noted for apple flesh. This result was in agreement with other studies, in which CV was shown to be an efficient instrumental tool for evaluating the reducing capacity of plant, food, and biological samples. An advantage of electrochemical measurement compared to DPPH and FRAP is that it is fast. CV measurement is carried out in <10 min, so it is less tedious. Moreover, it is not necessary to use expensive reagents that include free radicals, thus lowering the cost. In addition, the use of a small amount of organic solvent reduces the amount of organic waste produced.

### 3.4. Profile and Content of Phenolic Compounds in Apple Flesh and Peel Measured by HPLC–DAD–ESI-MS/MS

Detailed knowledge of the polyphenol profile and content in apple cultivars is necessary in order to evaluate their antioxidant activity and potential beneficial health effects. A comprehensive qualitative analysis of the phenolic compounds of the studied apple peel and flesh samples was achieved by HPLC–DAD–MS/MS. The composition and concentrations of identified compounds in the peel and flesh are presented in Table 3. Eleven polyphenolic compounds belonging to five major groups were identified: chlorogenic acid (hydroxycinnamic acid), phloretin and phloridzin (dihydrochalcones), catechin and epicatechin (flavonols), and quercetin, and four derivatives (flavonols) and cyanidin 3-galactoside (anthocyanins). It was found that phenolic acids and flavonols were the two main groups of polyphenols identified in the studied apple cultivars, as previously reported [53]. Concentrations of individual phenolic compounds in peel and flesh identified by HPLC–DAD–MS/MS are shown in Table 3.

Among flavan-3-ols, epicatechin was the major compound of this group in apple peel and flesh. The highest concentration of epicatechin in peel was found for Quinte (297.77 µg/g FW) and Early Geneva (278.11 µg/g FW), while Delikates had the lowest content (94.79 µg/g FW). In the flesh, the Quinte cultivar was also found to possess the highest concentration of epicatechin (325.04 µg/g FW), followed by Early Geneva and Paulared (270.76 and 221.56 µg/g FW, respectively). These results are in accordance with those reported by Tsao et al. [13] for eight apple cultivars, in which epicatechin ranged from 17.9 to 591.6 µg/g FW in the peel and 16.0 to 142.3 µg/g FW in the flesh. Catechin was present in smaller amounts in peel extracts (3.83 to 92.16 µg/g FW) as well as flesh extracts (1.64 to 75.90 µg/g FW). These data are also in agreement with those obtained for eight traditional apple cultivars of Southern Italy [50], for which the concentration of catechin ranged from 0 to 76.7 µg/g FW).

Chlorogenic acid, which is the major compound of hydroxycinnamic acids, was mostly located in the apple flesh, except for Gloster, Jonagored, and Ligol cultivars, where the content of chlorogenic acid in peel was higher than in flesh. In the flesh, chlorogenic acid levels ranged between 18.21 and 451.53 µg/g FW, with the highest amounts being recorded in Papierówka, followed by Quinte, Antonówka, and Rubinola (Table 3). The lowest amounts were found in Delikates and Gloster (20.01 and 18.21 µg/g FW, respectively). The concentrations of chlorogenic acid obtained for some cultivars tested in this work, particularly Papierówka, were twice those found in the cultivars studied by Khanizadeh et al. [53] and Panzella et al. [50]. These observations indicate that the range of differences between the polyphenol profiles of apples are highly cultivar dependent.

Differences were also observed in the content of dihydrochalcones. Phloridzin (phloretin 2ʹ-glucoside) was the predominant dihydrochalcone found and identified in all tested apple peel and flesh extracts. Phloretin and phloretin derivatives have occasionally been found in apple in trace amounts [13]. Phloridzin concentration was higher in apple peel, with a mean value of 33.28 µg/g FW compared to 16.38 µg/g FW in flesh (Table 3). Khanizadeh et al. [53] reported an average concentration of phloridzin in the peel and flesh of 10.4 and 55.4 µg/g FW, respectively. Among the tested apple cultivars, Papierówka in particular was characterized by the highest level of phloridzin (84.10 µg/g FW in peel), whose anti-diabetic properties have recently been reported by Masumoto et al. [54]. Even though dihydrochalcones exist in relatively low amounts due to the uniqueness of the apple and their different profiles among different cultivars, they have been used to distinguish apple from a number of other fruits and to identify apple cultivars [13].

Flavonols represent the second largest group in terms of concentration in apple peel. These polyphenols were constituted mainly by quercetin 3-arabinoside, followed by 3-rhamnoside and 3-glucoside, and slightly by quercetin and rutin. Depending on the cultivar, the total flavonols in the peel varied from 193.09 to 808.25 µg/g FW, with Sunrise showing the highest concentration (Table 3). These data are consistent with those reported by Tsao et al. [13]. On the other hand, in the flesh extracts of studied apple cultivars, only small amounts of quercetin 3-arabinoside, 3-rhamnoside, and 3-glucoside were detected (Table 3).

The major anthocyanins in apple are cyanidin glycosides, among which 3-galactoside is the predominant individual compound. Anthocyanins were found only in apples characterized by red and partially red skin (Quinte, Paulared, Gloster, and Rubinola), and only cyanidin 3-galactoside was identified in our study (Table 3). Cyanidin 3-galactoside content ranged from 1.15 to 103.69 µg/g FW and was the highest for Rubinola. This observation was consistent with that reported by Khanizadeh et al. [53].

According to Kschonsek et al. [43], the most abundant flavonoids that occur in apples (raw, with skin) are (-)-epicatechin, (+)-catechin, and cyanidin. The same flavonoids were found in apples without skin, but also a high amount of (-)-epigallocatechin. The main polyphenols that can be found in apples are quercetin, (-)-epicatechin, (+)-catechin, procyanidines, and anthocyanidines; dihydrochalcones; phloretin and phloridzin derivatives; and other phenolic compounds, such as chlorogenic acid. In addition, it was important that apples were shown to have the highest portion of free phenolics when compared to other fruits [4]. Apple’s bound phenolics have lower bioavailability as compared to free phenolics since they need to be released from the food matrix after digestion [55].

### 3.5. Antioxidant, Reducing, and Chelating Activities of Phenolic Compounds in Apple Flesh and Peel

The antioxidant, reducing, and chelating activities of phenolic compounds identified in apple peel and flesh by HPLC–DAD–MS/MS are shown in Table 4. The antioxidant activity of phenolic compounds from apple flesh and peel, determined as free radical-scavenging activity against stable, nonbiological relevant DPPH radicals, is expressed as Trolox equivalent. As it was defined, antioxidant activity is equal to the millimolar concentration of a Trolox solution that has antioxidant capacity equivalent to a 1.0 mM solution of the substance under investigation.

Quercetin, cyanidin 3-galactoside, rutin, catechin, and chlorogenic acid (2.09–1.45 mM Trolox) showed the highest ability to scavenge DPPH radicals, followed by quercetin 3-glucoside, epicatechin, quercetin 3-rhamnoside, and quercetin 3-arabinoside (1.42–0.95 mM Trolox), while the ability to scavenge DPPH radicals by phloretin (0.19 mM Trolox) and phloridzin (0.06 mM Trolox) was the lowest.

The order of reducing activity as shown by the FRAP assay was as follows: cyanidin 3-galactoside > quercetin > chlorogenic acid > quercetin 3-glucoside > catechin > epicatechin > quercetin 3-rhamnoside > rutin > quercetin 3-arabinoside > phloretin > phloridzin (5.69–0.18 mM Trolox). This order was supported by a study on the structure–activity relationship (SAR) of flavonoids [56,57].

**Figure 3:**
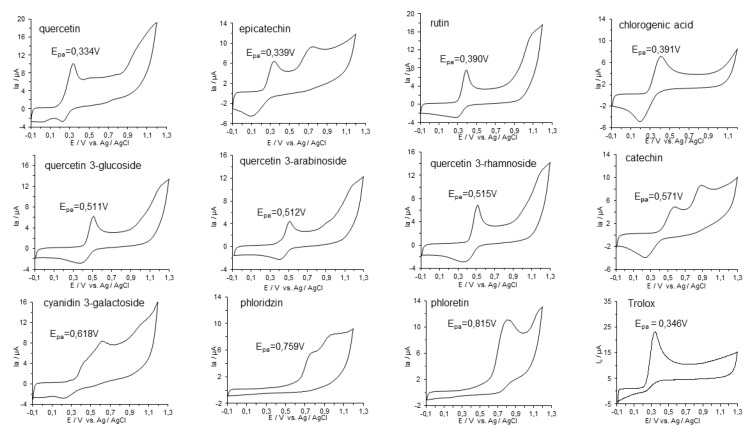
Cyclic voltammograms of 0.25 mM of standard solutions (final concentration) of phenolic compounds in apple cultivars identified by HPLC–DAD–MS/MS analysis in Britton–Robinson (B–R) buffer (0.1 M; pH 7.4) recorded from –100 to +1300 mV; scan rate 100 mV s^−1^.

In this study, cyclic voltammograms of the phenolic compounds identified in apple flesh and peel were recorded in the range of –100 to +1300 mV at a scanning rate of 100 mV s^−1^. Cyclic voltammograms of 0.25 mM solutions of examined compounds in 0.1 M Britton–Robinson (B–R) buffer (pH 6.0) in 80% methanol are shown in Figure 3.

The cyclic voltammograms showed that all compounds had well defined reversible waves with the first oxidation peak potential. The first anodic peak potential (E_pa_) of the investigated compounds varied according to the following gradation: phloretin (0.815 V) > phloridzin (0.759 V) > cyanidin 3-galactoside (0.618 V) > catechin (0.571 V) > quercetin 3-rhamnoside (0.515 V) > quercetin 3-arabinoside (0.512 V) ≥ quercetin 3-glucoside (0.511 V) > chlorogenic acid (0.391 V) ≥ rutin (0.390 V) > epicatechin (0.339 V) ≥ quercetin (0.334 V) as compared to Trolox (0.346 V). Higher Ep_a_ values were associated with lower reducing activity of the tested compound. Therefore, taking into account the values of the first oxidation potential of the studied compounds, almost all phenolic compounds identified in apple flesh and peel can be described as having high (intermediate) antioxidant power (Ep < 0.8 V), while phloretin had low antioxidant power (0.8 V < Ep < 1.3 V). This conclusion was drawn based on the work by Blasco et al. [58], in which the differentiation of the antioxidant power of phenolic compounds was based on their oxidation potential. When the calculation of antioxidant activity was based on the area under the anodic current waveform within the range of 0 to 1100 mV for each compound and Trolox, then the order of antioxidant activity was as follows: quercetin > epicatechin > cyanidin 3-galactoside > rutin ≥ phloretin > catechin > chlorogenic acid > phloridzin ≥ quercetin 3-rhamnoside ≥ quercetin 3-glucoside > quercetin 3-arabinoside (0.90–0.19 mM Trolox). The gradation of samples for reducing activity as determined by CV mirrored that obtained with the FRAP assay.

The highest ferrous ion chelating activity was shown by chlorogenic acid (88.47%), followed by rutin, quercetin, catechin, epicatechin, quercetin 3-glucoside, quercetin 3-arabinoside, and quercetin 3-rhamnoside (68.13%); activity twice as low was noted for cyanidin 3-galactoside, and the lowest was for phloridzin and phloridzin (5.68 and 1.15%, respectively).

### 3.6. Phenolic Contribution to Antioxidant Activity

A correlation analysis was performed to assess the contribution of polyphenolic compounds to antioxidant capacity. Interestingly, the association between antioxidant assays and the content of bioactive compounds differed between apple peel and flesh (Table 5).

In apple peel, catechin and epicatechin content was positively correlated to FRAP assay, while cyadinin-3-galactoside was highly associated with DPPH. Moreover, a strong correlation was found between the total phenolic and flavonoid content and FRAP and CV (Table 5).

In the apple flesh, FRAP showed a very strong correlation with epicatechin, chlorogenic acid, and cyaniding-3-galactoside. Similarly, DPPH and CV were correlated with the same compounds, and CV was additionally correlated with catechin. The chelating activity assay showed the strongest correlation with phloretin, phloridzin, and chlorogenic acid (Table 5). Among the antioxidant capacity tests, the strongest correlation was observed between CV and FRAP in apple peel, and between all assays in apple flesh.

## 4. Conclusions

It needs to be noted that the ripe apples are a good source of phenolic compounds, being present in both peel and flesh. According to the available literature, the geographical origin and variety of apples influence the content of phenolic compounds and are highly related to their antioxidant activity fluctuation. In this study, the apple peel and flesh of early varieties such as Antonówka, Delikates, Early Geneva, Papierówka, Paulared, Sunrise, Quinte showed higher total phenolic and flavonoids content as compared to the late varieties such as Gloster, Jonagored, Ligol and Rubinola. The HPLC–DAD–MS/MS analysis showed that the dominant compounds were catechin, epicatechin, chlorogenic acid, quercetin 3-glucoside, quercetin 3-arabinoside, quercetin 3-rhamnoside, cyanidin 3-galactoside and phloridzin whereas phloretin, quercetin and rutin were present in low concentration. These phenolic compounds varied considerably among apple cultivars and their content was higher in peels than in flesh. Information about cultivar--typical apple polyphenol content and profile is important for bioactivity studies and, consequently, essential for the development of consumer-relevant products with particular nutritional functionalities. Therefore, it can be concluded that whole ripe apples should be used as a relevant source of phenolics in our diet since the removal of peel from apple may induce a significant loss of antioxidants. On the other hand, apple peels can be a good component to formulate functional foods after currently proposed “cold-pressing technology” as an effective method for peeling and deseeding apple fruits, with a positive effect on phenolic compounds retention in pomace. This study showed that the application of HPLC–DAD–MS/MS analysis of phenolic compounds with the spectrophotometric methods for the determination of their antioxidant, reducing and chelating capacity and their electroactivity provided by cyclic voltammetry was essential to show their contribution to the antioxidant capacity of apple peel and flesh. This study also provides evidence to support the application of cyclic voltammetry as a rapid method in determining the phenolic profile and reducing power of apple flesh and peel.

## Figures and Tables

**Table 1:** MS data of phenolic compounds from apple.

Identification	(M)^−^ (*m*/*z*)	(M)^+^ (*m*/*z*)	MS/MS (*m*/*z*)
Cyanidin 3-*O*-galactoside		449.0	287.0
Phloretin	273.1		227.1/166.8/123.1
Phloridzin	435.2		273.1
Catechin	289.2		245.3/203.1/109.1
Epicatechin	289.2		245.3/203.1/109.1
Chlorogenic acid	353.2		191.1/179.1
Rutin	609.0		463.0/301.0
Quercetin 3-*O*-glucoside	463.0		301.0
Quercetin	301.0		179.0/151.0
Quercetin 3-*O*-arabinoside	433.0		301.0
Quercetin 3-*O*-rhamnoside	447.0		301.0

**Table 2:** Total phenolic content (TPC), total flavonoid content (TFC), Fe^2+^ chelation activity, and antioxidant activity by ferric-reducing/antioxidant power assay (FRAP), 2,2-diphenyl-1-picrylhydrazyl (DPPH), and cyclic voltammetry (CV) of the peel and flesh of different apple cultivars.

Apple Cultivars	FRAP	DPPH	CV	Chelating Activity	TPC	TFC
**Apple Peel**						
Quinte	21.31 ± 0.06a	7.51 ± 0.02d	6.09 ± 0.26b, c	51.80 ± 0.43b	3278.6 ± 29.0a	970.6 ± 6.3b
Jonagored	20.89 ± 0.23b	8.65 ± 0.02a	6.37 ± 0.32a, b	30.00 ± 0.39g	3123.1 ± 30.6b	830.1 ± 3.5e
Early Gen.	20.20 ± 0.21c	7.14 ± 0.01f	5.75 ± 0.15^c^	48.23 ± 0.58d	3147.0 ± 38.1b	835.2 ± 23.3e
Paulared	19.38 ± 0.20d	7.39 ± 0.03e	6.80 ± 0.33a	39.90 ± 0.58e	2916.1 ± 42.5c	1303.8 ± 14.2a
Sunrise	17.44 ± 0.11e	6.90 ± 0.02g	5.86 ± 0.39c	36.36 ± 0.31f	2716.3 ± 37.2d	905.8 ± 5.6c
Gloster	16.90 ± 0.10f	8.19 ± 0.00b	5.69 ± 0.16c	24.27 ± 0.18h	2517.2 ± 56.2e	696.7 ± 3.1h
Delikates	14.70 ± 0.16g	6.67 ± 0.03h	5.23 ± 0.15d	30.71 ± 0.28g	2333.7 ± 16.1f	628.2 ± 10.9i
Papierówka	14.30 ± 0.16h	5.62 ± 0.03i	5.13 ± 0.31d	49.98 ± 0.88c	2194.7 ± 9.4g	875.1 ± 6.5d
Rubinola	14.21 ± 0.11h	7.91 ± 0.01c	4.97 ± 0.25d	23.59 ± 0.27h	2296.9 ± 4.1f	714.6 ± 2.4g
Antonówka	12.73 ± 0.23i	5.27 ± 0.03j	4.35 ± 0.22e	53.54 ± 0.61a	2051.6 ± 21.9h	766.6 ± 8.0f
Ligol	12.40 ± 0.14j	6.87 ± 0.02g	4.46 ± 0.15e	19.30 ± 0.36i	1821.3 ± 20.7i	553.1 ± 3.2j
**Apple Flesh**						
Quinte	10.15 ± 0.12a	4.65 ± 0.01a	3.95 ± 0.17a	45.89 ± 0.33d	1740.3 ± 19.4a	737.6 ± 3.9c
Jonagored	4.88 ± 0.07f	3.01 ± 0.01g	1.91 ± 0.04d	21.89 ± 0.12i	734.9 ± 7.2g	217.1 ± 1.9h, i
Early Gen.	9.28 ± 0.09b	4.20 ± 0.04b	3.14 ± 0.17b	47.51 ± 0.19c	1578.5 ± 33.3b	612.2 ± 9.2e
Paulared	7.51 ± 0.04d	3.48 ± 0.02d	3.04 ± 0.37b, c	33.52 ± 0.86e	1217.5 ± 4.1e	765.5 ± 6.6b
Sunrise	5.09 ± 0.09e	2.47 ± 0.02h	1.96 ± 0.22d	29.85 ± 0.23f	811.1 ± 11.1f	311.5 ± 4.0f
Gloster	3.15 ± 0.10i	2.23 ± 0.01k	1.49 ± 0.01e	23.02 ± 0.31h	544.3 ± 1.8i	223.0 ± 2.3h
Delikates	4.51 ± 0.05g	2.42 ± 0.01i	1.84 ± 0.25d	25.06 ± 1.12g	711.2 ± 7.3h	206.0 ± 6.3i
Papierówka	8.39 ± 0.02c	3.85 ± 0.01c	3.35 ± 0.20b	49.46 ± 0.54b	1356.2 ± 7.1c	831.1 ± 15.9a
Rubinola	4.80 ± 0.08f	3.12 ± 0.04f	1.92 ± 0.13d	24.99 ± 0.24g	830.4 ± 4.9f	296.9 ± 4.0g
Antonówka	7.60 ± 0.10d	3.19 ± 0.02e	2.83 ± 0.07c	60.0 ± 0.1a	1246.4 ± 13.4d	656.3 ± 5.9d
Ligol	3.44 ± 0.11h	2.37 ± 0.02j	1.44 ± 0.07e	18.31 ± 0.67j	535.6 ± 4.9i	178.7 ± 1.9j

Values represent the mean (*n* = 3) ± SD. Different letters a–j in the same column for peel or flesh indicate significant differences by ANOVA test (*p* < 0.05). Results for ferric reducing antioxidant power (FRAP), DPPH, and CV are expressed in Trolox equivalent per gram of apple fresh weight (μmol TE/g FW), and for chelating activity in % of chelating of Fe(II). TPC and TFC results are expressed in μg catechin equivalent (CAE)/g FW.

**Table 3:** Concentrations of the individual phenolic compounds identified by the HPLC–DAD–MS/MS analysis in the apple peel and flesh in different cultivars.

Cultivar											
	Quinte	Jonagored	Early Geneva	Paulared	Sunrise	Gloster	Delikates	Papierówka	Rubinola	Antonówka	Ligol
**Apple Peel**											
Phloretin	0.96 ± 0.02d	0.91 ± 0.08d	0.85 ± 0.02e	0.64 ± 0.01g	1.38 ± 0.08b	1.32 ± 0.02b	b0.73 ± 0.03f	2.25 ± 0.05a	1.24 ± 0.05c	1.20 ± 0.01c	0.66 ± 0.04g
Phloridzin	23.91 ± 0.48d	24.65 ± 1.63d	24.45 ± 0.49d	16.43 ± 0.76e	48.08 ± 0.80b	43.45 ± 2.59c	18.30 ± 1.74e	84.10 ± 1.60a	42.90 ± 3.21c	23.37 ± 0.45d	16.51 ± 0.73e
Catechin	92.16 ± 1.99a	7.93 ± 0.07h, i	28.56 ± 0.62d	53.66 ± 0.46b	41.34 ± 0.29c	7.32 ± 0.53i	3.83 ± 0.31j	17.93 ± 1.21e	10.56 ± 0.80g	12.40 ± 0.12f	8.58 ± 0.29h
Epicatechin	297.77 ± 0.91a	103.17 ± 5.97h	278.11 ± 15.55b	161.68 ± 10.26e	198.36 ± 5.41c	142.23 ± 1.43f	94.79 ± 4.34h	165.57 ± 12.39e	182.86 ± 5.22d	127.72 ± 8.40g	101.28 ± 6.79h
Chlorogenic acid	188.59 ± 9.98c	57.00 ± 6.46g	57.41 ± 2.45g	68.97 ± 4.14f	87.27 ± 2.24e	61.67 ± 0.74g	8.05 ± 0.36h	259.58 ± 3.19a	206.97 ± 1.09b	137.17 ± 2.99d	58.62 ± 1.08g
Rutin	14.54 ± 0.60f	15.33 ± 0.47e, f	3.36 ± 0.07h	16.52 ± 0.49e	73.03 ± 0.94a	27.55 ± 2.38c	12.21 ± 0.67g	2.97 ± 0.08h	22.97 ± 0.74d	32.35 ± 0.72b	10.59 ± 3.31g
Quercetin 3-glucoside	131.46 ± 2.06b	120.55 ± 1.62c	33.80 ± 5.66h	76.04 ± 0.48e	265.76 ± 0.56a	125.36 ± 2.05c	95.13 ± 7.38d	52.41 ± 1.69g	68.01 ± 4.92f	49.20 ± 3.38g	70.20 ± 4.40f
Quercetin	0.98 ± 0.01a	0.73 ± 0.00d, e	0.65 ± 0.01g	0.77 ± 0.00c	0.81 ± 0.01b	0.69 ± 0.00f	0.73 ± 0.00d	0.71 ± 0.00e	0.80 ± 0.01b	0.61 ± 0.02h	0.76 ± 0.03c
Quercetin 3-arabinoside	185.98 ± 0.69b	167.01 ± 1.68c	98.70 ± 2.20e	100.39 ± 2.96e	241.28 ± 8.75a	179.38 ± 14.91b	112.36 ± 0.94d	97.04 ± 0.98e	58.45 ± 0.88g	87.63 ± 5.37f	83.94 ± 3.32f
Quercetin 3-rhamnoside	97.45 ± 14.86d	112.52 ± 1.94c	90.41 ± 2.41d	40.72 ± 1.12h	222.37 ± 1.80b	114.83 ± 3.98c	74.87 ± 0.97f	39.96 ± 1.51h	235.08 ± 0.43a	51.05 ± 1.08g	82.41 ± 4.61e
Cyanidin 3-galactoside	99.16 ± 2.16b	45.93 ± 0.48e	31.21 ± 1.32f	63.82 ± 4.79c	18.00 ± 1.52g	101.92 ± 0.31a	49.20 ± 0.95d	1.15 ± 0.00h	103.69 ± 2.17a	1.24 ± 0.00h	30.69 ± 1.00f
**Apple Flesh**											
Phloretin	0.72 ± 0.03b	0.42 ± 0.01g	0.72 ± 0.01b	0.51 ± 0.02e	0.60 ± 0.03d	0.41 ± 0.00g	0.47 ± 0.01e, f	0.75 ± 0.03b	0.65 ± 0.03c	1.38 ± 0.08a	0.43 ± 0.01f, g
Phloridzin	18.89 ± 0.98d	7.17 ± 0.17h	20.63 ± 0.80c	10.37 ± 0.36f	14.68 ± 0.84e	5.86 ± 0.16i	9.14 ± 0.06g	23.46 ± 0.77b	18.09 ± 0.22d	45.05 ± 1.79a	6.61 ± 0.14h, i
Catechin	39.46 ± 0.20b	1.07 ± 0.01i	21.83 ± 0.61e	75.90 ± 1.44a	30.91 ± 0.49c	1.64 ± 0.57i	3.91 ± 0.22h	25.90 ± 0.33d	10.67 ± 0.39g	14.00 ± 0.29f	1.66 ± 0.06i
Epicatechin	325.04 ± 1.63a	18.64 ± 3.02g	270.76 ± 8.20b	221.56 ± 24.07c	68.76 ± 0.68f	4.99 ± 0.13h	68.27 ± 3.92f	154.17 ± 7.08d	100.67 ± 2.23e	78.23 ± 2.56f	13.81 ± 0.35g, h
Chlorogenic acid	307.17 ± 3.22b	43.56 ± 0.73g	154.91 ± 0.73e	265.59 ± 4.52d	85.07 ± 1.80f	18.21 ± 0.71h	20.01 ± 2.19h	451.53 ± 19.59a	264.47 ± 10.42d	293.56 ± 7.19c	26.74 ± 2.63h
Rutin	0.79 ± 0.00d	1.07 ± 0.05a	0.70 ± 0.01g	0.84 ± 0.00c	1.00 ± 0.00b	0.72 ± 0.01f	0.75 ± 0.00e	0.64 ± 0.00h	0.75 ± 0.01e	0.64 ± 0.00h	0.69 ± 0.03g
Quercetin 3-glucoside	2.12 ± 0.03a	1.58 ± 0.02c	1.27 ± 0.03e	1.97 ± 0.03b	1.96 ± 0.07b	1.06 ± 0.12g	1.34 ± 0.02d	1.06 ± 0.03g	1.03 ± 0.05g	0.81 ± 0.00h	1.18 ± 0.04f
Quercetin	n.d.	n.d.	n.d.	n.d.	n.d.	n.d.	n.d.	n.d.	n.d.	n.d.	n.d.
Quercetin 3-arabinoside	6.89 ± 0.17a	5.69 ± 0.45b	4.10 ± 0.08d	4.84 ± 0.19c	4.60 ± 0.40c	2.33 ± 0.25f	1.47 ± 0.05g, h	2.84 ± 0.21e	1.76 ± 0.15g	1.21 ± 0.01h	2.76 ± 0.02e
Quercetin 3-rhamnoside	1.63 ± 0.04g	15.39 ± 0.32a	1.12 ± 0.02i	1.38 ± 0.02h	10.55 ± 0.36b	3.01 ± 0.04f	1.72 ± 0.06g	3.68 ± 0.05e	4.67 ± 0.29d	1.15 ± 0.04h, i	6.22 ± 0.12c
Cyanidin 3-galactoside	1.29 ± 0.06a	0.68 ± 0.01e	1.10 ± 0.00b	0.83 ± 0.01c	0.70 ± 0.00d, e	0.59 ± 0.00g	0.72 ± 0.00d	0.54 ± 0.00h	0.65 ± 0.01f	0.55 ± 0.00h	0.61 ± 0.00g

Values represent the mean (*n* = 4) ± SD. Different letters a–j in the same row related to apple peel or flesh indicate significant differences by ANOVA test (*p* < 0.05). n.d., not detected. Results are expressed in μg/g FW.

**Table 4:** Antioxidant, reducing, and chelating activities of phenolic compounds identified in apple peel and flesh by HPLC–DAD–MS/MS.

Compound/Assay	Antioxidant Activity (mM Trolox)	Reducing Activity (mM Trolox)		Chelating Activity (%)
	DPPH	FRAP	CV	FZ
Phloretin	0.19 ± 0.01	0.95 ± 0.02	0.46 ± 0.02	1.15 ± 0.06
Phloridzin	0.06 ± 0.01	0.18 ± 0.01	0.26 ± 0.03	5.68 ± 0.30
Catechin	1.55 ± 0.02	1.97 ± 0.01	0.39 ± 0.03	74.56 ± 3.65
Epicatechin	1.37 ± 0.01	1.95 ± 0.15	0.69 ± 0.03	70.14 ± 2.80
Chlorogenic acid	1.45 ± 0.01	3.71 ± 0.07	0.34 ± 0.02	88.47 ± 2.65
Rutin	1.69 ± 0.02	1.64 ± 0.06	0.46 ± 0.01	85.33 ± 2.56
Quercetin 3-glucoside	1.42 ± 0.03	2.08 ± 0.01	0.23 ± 0.03	70.25 ± 2.10
Quercetin	2.09 ± 0.03	3.68 ± 0.19	0.90 ± 0.04	76.84 ± 2.31
Quercetin 3-arabinoside	0.95 ± 0.03	1.52 ± 0.01	0.19 ± 0.20	69.74 ± 2.10
Quercetin 3-rhamnoside	1.27 ± 0.02	1.89 ± 0.03	0.24 ± 0.01	68.13 ± 2.38
Cyanidin 3-galactoside	2.07 ± 0.03	5.69 ± 0.02	0.65 ± 0.02	29.11 ± 1.02

Results were provided by DPPH radical scavenging activity assay. FRAP: ferric-reducing/antioxidant power assay; CV: cyclic voltammetry assay; FZ: ferrozine assay. Data expressed as the mean ± standard deviation (*n* = 9).

**Table 5:** Correlation coefficient between phenolic compounds and antioxidant capacity tests and linear correlation coefficient between different methods for antioxidant capacity assessment in apple peel and flesh.

	FRAP	DPPH	CV	Chelating Activity
**Apple Peel**				
Phloretin	−0.189	−0.388	−0.232	0.295
Phloridzin	−0.155	−0.258	−0.134	0.156
Catechin	0.628 *	0.079	0.541	0.526
Epicatechin	0.659 *	0.083	0.332	0.549
Chlorogenic acid	−0.110	−0.312	−0.232	0.420
Rutin	−0.093	−0.015	0.045	−0.090
Quercetin-3-glucoside	0.198	0.270	0.362	−0.174
Quercetin	0.299	0.353	0.365	−0.029
Quercetin-3-arabinose	0.460	0.330	0.509	0.027
Quercetin-3-rhamnoside	−0.045	0.439	0.015	−0.439
Cyadinin-3-galactoside	0.190	0.744 **	0.350	−0.393
Total polyphenol	0.980 ***	0.553	0.867 **	0.324
Total flavonoid	0.607 *	0.069	0.752 **	0.498
FRAP	1.000	–	–	–
DPPH	0.413	1.000	–	–
CV	0.824 **	0.600	1.000	–
Chelating activity	0.451	−0.562	0.104	1.000
**Apple Flesh**				
Phloretin	0.551	0.372	0.491	0.862 **
Phloridzin	0.590	0.412	0.519	0.879 ***
Catechin	0.524	0.508	0.642 *	0.340
Epicatechin	0.874 ***	0.915 ***	0.897 ***	0.587
Chlorogenic acid	0.773 **	0.719 *	0.791 **	0.737 *
Rutin	−0.272	−0.174	−0.225	−0.444
Quercetin-3-glucoside	0.203	0.282	0.321	−0.122
Quercetin	–	–	–	–
Quercetin-3-arabinose	0.379	0.521	0.455	0.020
Quercetin-3-rhamnoside	−0.447	−0.360	−0.466	−0.508
Cyadinin-3-galactoside	0.604 *	0.720 *	0.626 *	0.267
Total polyphenol	0.988 ***	0.956 ***	0.978 ***	0.838 **
Total flavonoid	0.905 ***	0.820 **	0.939 ***	0.841 **
FRAP	1.000	–	–	–
DPPH	0.943 ***	1.000	–	–
CV	0.975 ***	0.939 ***	1.000	–
Chelating activity	0.866 **	0.694 *	0.816 **	1.000

*, **, *** Significant correlation at *p* < 0.05, *p* < 0.01, *p* < 0.001, respectively.
